# Regional perturbation of gene transcription is associated with intrachromosomal rearrangements and gene fusion transcripts in high grade ovarian cancer

**DOI:** 10.1038/s41598-019-39878-9

**Published:** 2019-03-05

**Authors:** Paul M. Krzyzanowski, Fabrice Sircoulomb, Fouad Yousif, Josee Normand, Jose La Rose, Kyle E. Francis, Fernando Suarez, Tim Beck, John D. McPherson, Lincoln D. Stein, Robert K. Rottapel

**Affiliations:** 1 Department of Medicine, University of Toronto, Ontario Institute for Cancer Research, MaRS Centre, Toronto, Ontario Canada; 2 Department of Immunology, University of Toronto, Princess Margaret Cancer Center, MaRS Centre, Toronto, Ontario Canada; 3 Department of Medical Biophysics, University of Toronto, Dalhousie University, Halifax, Nova Scotia Canada; 4 Human Longevity Inc., San Diego, California USA; 5 University of California, Davis Medical Center, Sacramento, California USA; 6 Department of Molecular Genetics, University of Toronto, Toronto, Ontario Canada

## Abstract

Genomic rearrangements are a hallmark of cancer biology and progression, allowing cells to rapidly transform through alterations in regulatory structures, changes in expression patterns, reprogramming of signaling pathways, and creation of novel transcripts via gene fusion events. Though functional gene fusions encoding oncogenic proteins are the most dramatic outcomes of genomic rearrangements, we investigated the relationship between rearrangements evidenced by fusion transcripts and local expression changes in cancer using transcriptome data alone. 9,953 gene fusion predictions from 418 primary serious ovarian cancer tumors were analyzed, identifying depletions of gene fusion breakpoints within coding regions of fused genes as well as an N-terminal enrichment of breakpoints within fused genes. We identified 48 genes with significant fusion-associated upregulation and furthermore demonstrate that significant regional overexpression of intact genes in patient transcriptomes occurs within 1 megabase of 78 novel gene fusions that function as central markers of these regions. We reveal that cancer transcriptomes select for gene fusions that preserve protein and protein domain coding potential. The association of gene fusion transcripts with neighboring gene overexpression supports rearrangements as mechanism through which cancer cells remodel their transcriptomes and identifies a new way to utilize gene fusions as indicators of regional expression changes in diseased cells with only transcriptomic data.

## Introduction

High grade serous ovarian cancer (HGSOC) is the second most common gynecological cancer and the most aggressive form of ovarian cancer^1^. HGSOC accounts for just 3% of all cancers in women, but is among the most lethal with a five year survival rate of only 20% and an overall mortality rate of 70%^2^. HGSOC is a disease of the damaged genome, characterized by widespread copy number alterations, a high frequency of TP53 mutations, but a paucity of dominant acting oncogenes^3^. In addition to chromatin damage surveillance defects due to TP53 inactivation, HGSOC has a high frequency of inactivating mutations in other DNA damage repair pathways; it has been estimated that somatic alterations in BRCA1/2 and other Homologous Recombination Deficiency (HRD) genes may affect a total of 50% of HGSOC patients^4,5^. HGSOC is characterized with gains and losses of chromosome arms or entire chromosomes, and is amongst the most genomically unstable tumors of those that have been studied to date with a median 33% of the genome undergoing copy number alterations^6^. In addition, a recent pan-cancer survey of whole genomes revealed that inter- and intra-chromosomal rearrangements are common in HGSOC, with an average of 261 genomic breakpoint events per sample^7^. Extensive chromosomal disruption has been associated with poor prognosis^8^, presumably due to the ability of genetically unstable cells to develop resistance to chemotherapy. High rates of chemoresistance may also be a reflection of the high levels of intratumoral genetic heterogeneity that is a hallmark of this cancer^9^. Because of the centrality of HGSOC’s genomic instability to its biology and clinical behavior, understanding the consequences of its genomic instability is essential to developing more effective biomarkers and therapeutic targets.

### Relevance of gene fusions to ovarian cancer

The high level of genomic instability in HGSOC creates a fertile environment for the acquisition of fusion genes which can create neomorphic cancer drivers through the creation of novel domain combinations^10^. Intact domains in expressed protein fusions, in turn, can be selectively targeted^11,12^.

We sought to elucidate how expressed gene fusion events in HGSOC tumors might contribute to the transformed state. We demonstrate that in contrast to the traditional model of positive selection for fusion transcripts generating novel proteins, the majority of the fusion transcripts detected do not result in in-frame fusion transcripts, and that a subset of genes involved in fusion events instead exhibit transcriptional upregulation. We further identify a novel phenomenon in which expressed fusion events are frequently associated with perturbation in gene expression in the neighborhood of the fusion event, an effect that can extend up to a megabase from the site of rearrangement, consistent with other studies linking more comprehensive genomic data such as whole exome sequencing^13^, Single Nucleotide Polymorphism (SNP) array data^14^, or whole genome sequencing^15^ with altered expression of nearby genes. Our study utilizes transcriptome data alone to demonstrate remodeling of the transcriptome in HGSOC through gene fusion events that may contribute to the overall fitness of the evolving tumor.

## Results

### Expressed fusion transcripts are ubiquitous in ovarian cancer

**Figure 1:**
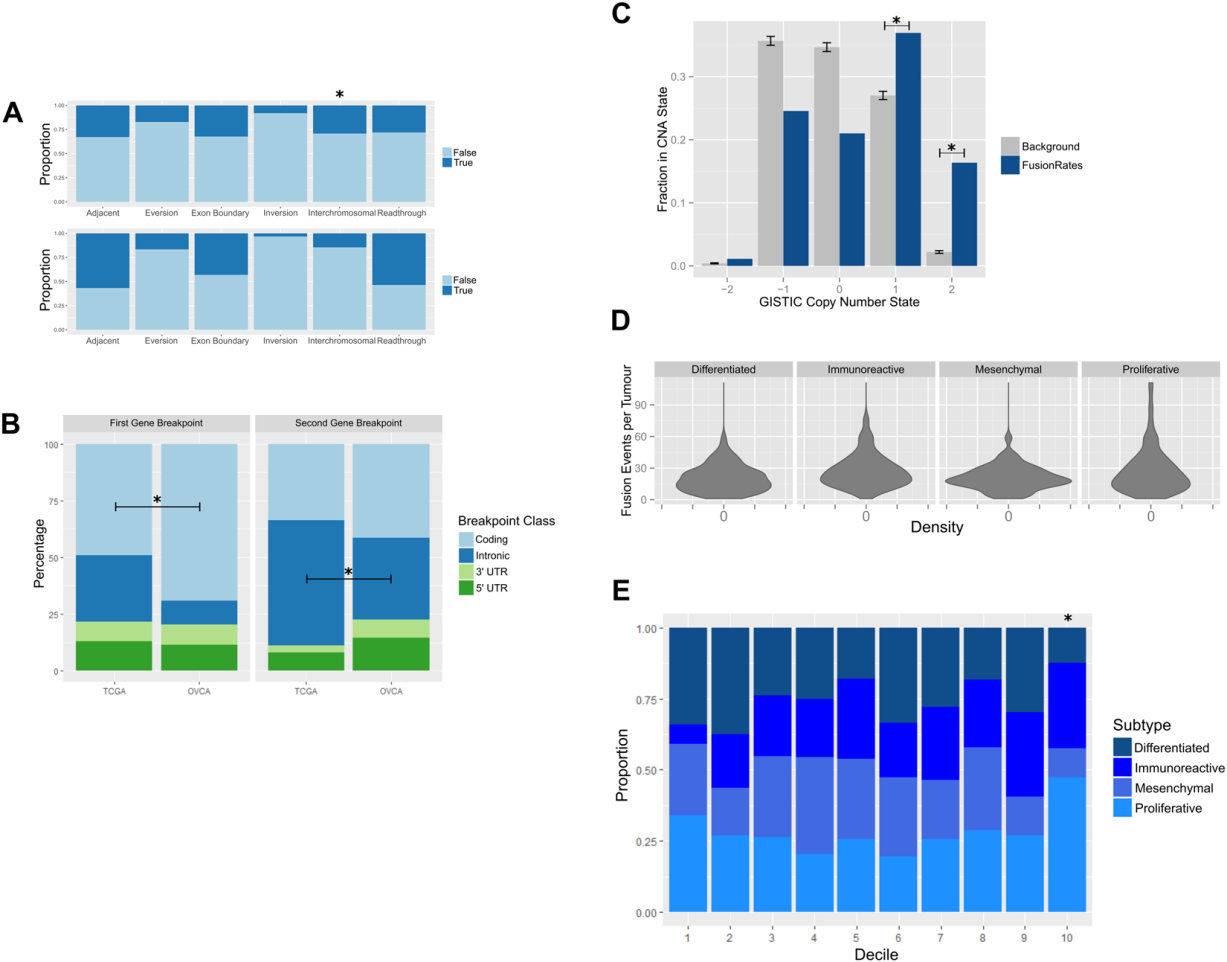
Characteristics of chimeric transcripts in ovarian cancer, in transcriptomes derived from TCGA primary tumors and ovarian cell lines. (**A**) Rearrangement classifications associated with fusion events, in TCGA primary transcriptomes and ovarian cancer cell lines. (**B**) Profiles of cDNA positions associated with expressed fusion transcripts in TCGA primaries. (**C**) Copy number status associated with evidence of TCGA fusion transcripts. (**D**) Density plots of fusion counts per tumor, separated by TCGA subgroups. (**E**) Proportions of OVCA subtypes vs expressed fusion load.

To characterize the landscape of expressed fusion transcripts, 420 HGSOC primary transcriptomes from The Cancer Genome Atlas (TCGA) project were analyzed using *defuse*, a package for prediction fusion transcripts from RNA sequencing (RNA-seq) data^16^. Analysis of 418 out of 420 transcriptomes were completed successfully, yielding 38,043 raw fusion predictions. Events also predicted using normal ovarian fallopian tube secretory epithelial cell samples were considered false positives and removed from the dataset (See Methods), leaving 9,953 fusion predictions (Supplementary Table 1). The number of expressed fusion events per tumor ranged from 1 to 111, with a median burden of 21 fusion events per case (Interquartile Range (IQR) = 14–30) (Fig. 1A). This set of predictions was used throughout this study.

To supplement this information, we performed RNA-seq on a set of 42 commonly used ovarian cancer cell lines. These lines included both serous (n = 33) and clear cell (n = 6) carcinomas, as well as cell lines of other cellular origins. Applying the same fusion prediction and filtering methods as used for the TCGA primaries, we made 3,037 raw fusion predictions, yielding 1,866 high confidence fusion transcripts after filtering (Supplementary Table 2). The fusion burden in cell lines was higher than in the primary tumors, with a median 38 chimeric transcripts expressed per cell line (range = 5–123, IQR = 23–60). The difference may be explained by sampling bias related to differences in clonality between pure populations of cultured cell lines and heterogeneous primaries, where putative fusion expressing subclones may be present in small fractions which may reduce fusion transcript abundance below limits of detection. Primary tumors in ovarian cancer are typically a mixture of tumor and stromal cells with median stromal proportions of 50%^17^.

### Mutational profile of ovarian fusion events in TCGA

We analyzed the structural features of this set of fusion transcripts (Fig. 1). Approximately 33% of detected fusions involved adjacent genes, which were predominantly classified as read-through transcripts (Fig. 1A). Mechanisms capable of generating read-through transcripts include rearrangements of genomic DNA, deletions of intergenic regions and mutations impacting transcriptional termination-events. The abundance of fusions involving adjacent genes in primary tumors (33%) is lower than the rate originally observed in ovarian cancer cell lines by McPherson *et al*. (68%, 1741/2540)^16^ and in the Australian Ovarian Cancer Study (AOCS) data (63%, 2050/3279), suggesting that either primary ovarian samples experience events that disrupt transcriptional termination less frequently than cell lines, or that a subset of fusion events are present at low abundance due to tumor heterogeneity and therefore not detected at a comparable level of sensitivity. Cell lines had higher rates of predicted read-through transcripts (54%; Fig. 1A) than the primary tumors, although still lower than previously reported in the literature.

One of the most striking features of both the primary and cell line data was the high observed rate of intrachromosomal fusions (70.8%; Fig. 1A). To determine the enrichment level of these events, the fusion generation process was modeled as a stochastic process of chromosome breakage and repair, leading to the observation that this rate was 14-fold higher in the primary tumor than would be expected (Permutation test P < 10^−5^). The rate of intrachromosomal fusion events was even higher among the sequenced ovarian cell lines (85.4%; Permutation test P < 10^−5^). These results contrast with the 11% intrachromosomal rate of breakpoints reported by ChimerDB for all types of biological samples^18^. In addition, ovarian cancer cell lines exhibited a higher proportion of breakpoints within coding regions than in primary tumor samples (Two-sided 4-sample proportionality test P-value < 10^−15^, Fig. 1B) and were enriched in breakpoints within the 5′-UTRs of the 3′ fusion gene partners (Two-sided 2-sample proportionality test P-value = <10^−15^).

Somatic copy number alterations (CNAs) are a known mechanism through which tumors can control dosage of tumor suppressors and oncogenes. We investigated the relationship between these events and fusions using TCGA data computed with GISTIC, a method for identifying somatic copy-number alterations (Fig. 1C)^19^. When copy number states for genes involved in fusions were compared both low and high level amplifications were found to be more frequently associated with fusions than with the background distribution of copy number levels, (Permutation Test, P < 1 × 10^−6^), confirming the previously-identified association of copy number events with chromosomal rearrangements^8^. However, our results indicate that the emergence of expressed fusion transcripts is not solely limited to regions of copy number gain.

Finally, we investigated whether the wide range of fusion burdens observed in ovarian patients were related to molecular subtypes (differentiated; immunoreactive; mesenchymal; and proliferative) as assigned by Verhaak *et al*. using CLOVAR, a single-sample gene set enrichment analysis (ssGSEA) classification approach^20^. Tumors classified as immunoreactive or proliferative tended to carry higher levels of expressed fusions compared to differentiated or mesenchymal cases (Fig. 1D). The enrichment for these subtypes was statistically significant in the highest decile of fusion burden (Fig. 1E, Fisher’s Exact Test, P = 5 × 10^−3^), suggesting that immunoreactive and proliferative subtypes of ovarian tumors have higher rates of genomic rearrangements.

### Gene fusions are enriched in N-terminal domains

Oncogenic fusion genes frequently coopt signaling proteins through truncation of their autoinhibitory domains as seen with ALK, ROS or RET fusions^21^. In these gene fusions, the secondary partner proteins typically participate in a passive role. In other cases, secondary partner proteins can create neomorphic functions of fusion oncogenes through the addition of subcellular localization sequences or dimerization domains of the primary fusion partner, as in forced homotypic dimerizations of RARα^22^. Thus, the need for driver gene domains to remain intact suggests that breakpoints might be biased towards the 5′ or 3′ end of the original coding transcript.

**Figure 2:**
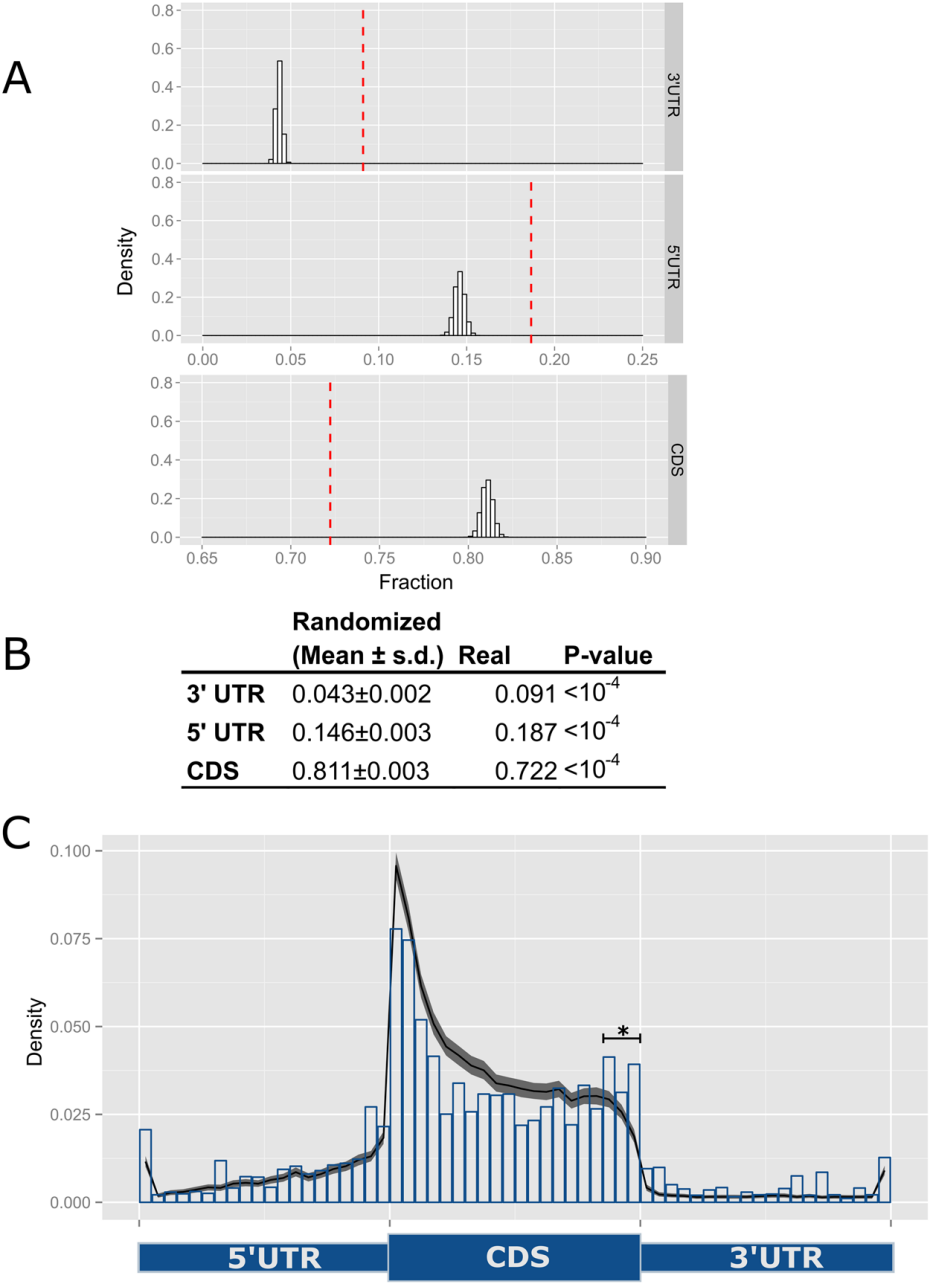
Gene fusion breakpoint locations within protein coding transcripts. (**A**) Expected distributions of cDNA breakpoint positions according to a random genomic breakage model. Red thresholds indicate observed positions. (**B**) Numerical data for A. (**C**) Normalized density plot illustrating observed breakpoint positions within cDNAs (bar plot) vs expected distribution (ribbon plot) with enrichment within C-terminal breakpoints of the CDS (star; Permutation test P < 10^−4^).

We first investigated whether bias for or against breakpoint position was detectable in each major region of mRNAs (Fig. 2A). We observed that fusion transcripts were significantly enriched in breakpoints residing in the 5′ or 3′ UTR regions of one or both participating transcripts (Fig. 2A,B, P < 10^−4^ by permutation test) and a significant depletion in breakpoint frequency within coding regions (P < 10^−4^ by permutation test). These observations suggest selection against fusion transcripts encoding neomorphic proteins, possibly directed towards a reduction in the creation of novel immunogenic proteins, which would be created through novel peptide sequences encoded across fusion junctions in RNA. Though immunogenic proteins can come from both in-frame and out-of-frame fusion proteins, frameshifted proteins should have a higher likelihood of being immunogenic through the production of novel amino acid sequences. To test this, we examined whether tumors with high and low proportions of out-of-frame fusions differed in regard to evidence of T-cell infiltration using a T-cell transcriptional signature (CD3D; CD3E; CD3G; CD6; SH2D1A; TRAT1)^23^, expecting to observe clustering of samples with high proportions of out-of-frame fusions according to this signature. However, we observed no clustering of tumors with high versus low burdens of out-of-frame fusion transcripts when using this signature (data not shown). These data suggest that evasion of immune selection pressure does not contribute to the paucity of frame-shifted fusion events.

Next, we tested for positional bias within the coding regions of genes involved in fusions. For example, if positive selection exists for fusions joining mostly intact coding regions of participating genes, we expected to observe an enrichment of breakpoints near the N- and C-termini of coding regions. Indeed, we found that TCGA ovarian fusion genes are enriched in breakpoints within the C-terminal region of coding sequences (P-value = 0.028, two-sample Kolmogorov-Smirnov (KS) test; Fig. 2C), but not the N-terminal portion (data not shown). Interestingly, the lack of enrichment within the N-terminus of coding regions may be due to the already high expected rate of breakpoints in this region, which is likely due to the 5′-bias observed in introns of eukaryotic genomes^24,25^. Together, these observations argue for the selection of events that lead to transcripts encoding fusion proteins with a predominantly intact, and at least partially functional, protein coding genes.

### The presence of fusion transcripts mark regions of dysregulated gene expression

Gene fusion events involved ‘promoter swapping’ often increase the expression of partner genes with otherwise low expression levels^26,27^. In addition to promoter swapping, genomic rearrangements can bring enhancers into new regions of the genome, which can alter the transcriptional landscape. We hypothesized that if a generalized process of rearranging active regulatory elements in ovarian cancer cells exists, at least one gene partner in ovarian fusion transcripts should frequently be identified to be highly active in normal ovarian tissues.

To test this, we investigated whether genes associated with detected ovarian fusions were enriched in highly expressed ovarian tissue genes defined by GTEx. GTEx ovarian genes were ranked by expression level and the top *n* highly expressed genes were considered ‘active’. Statistical enrichment for these highly expressed genes was observed for all sets with at least 200 genes (Cumulative hypergeometric test P < 10^−6^ in all cases). These results indicate that ovarian cancer related gene fusions preferentially involve genes normally expressed at high levels in ovarian tissues.

**Figure 3:**
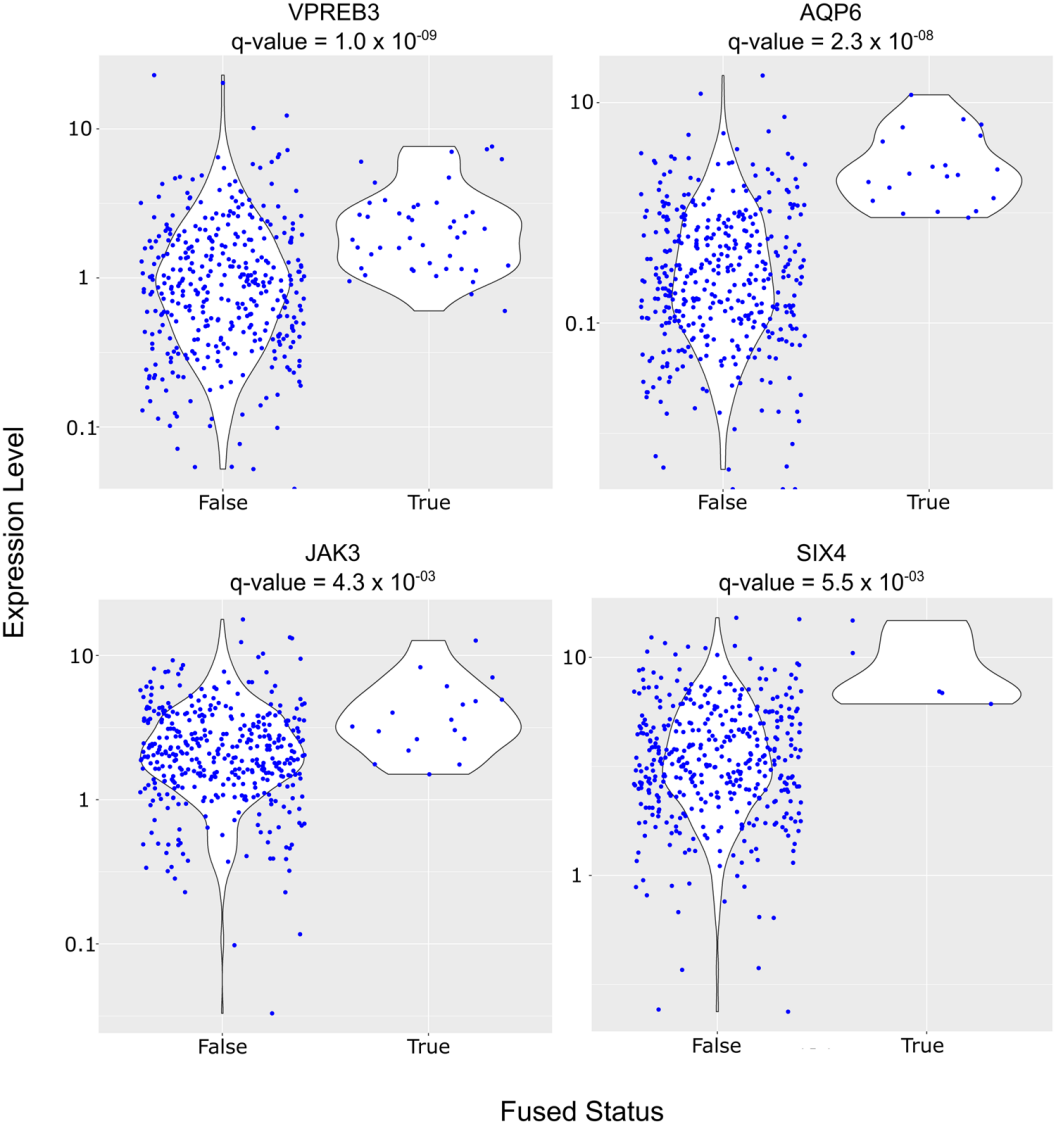
Selected genes with expression changes significantly correlated with their fusion status in primary ovarian cancer tumors. Expression levels (FPKM) shown on y-axis with fusion status presented on x-axis.

**Figure 4:**
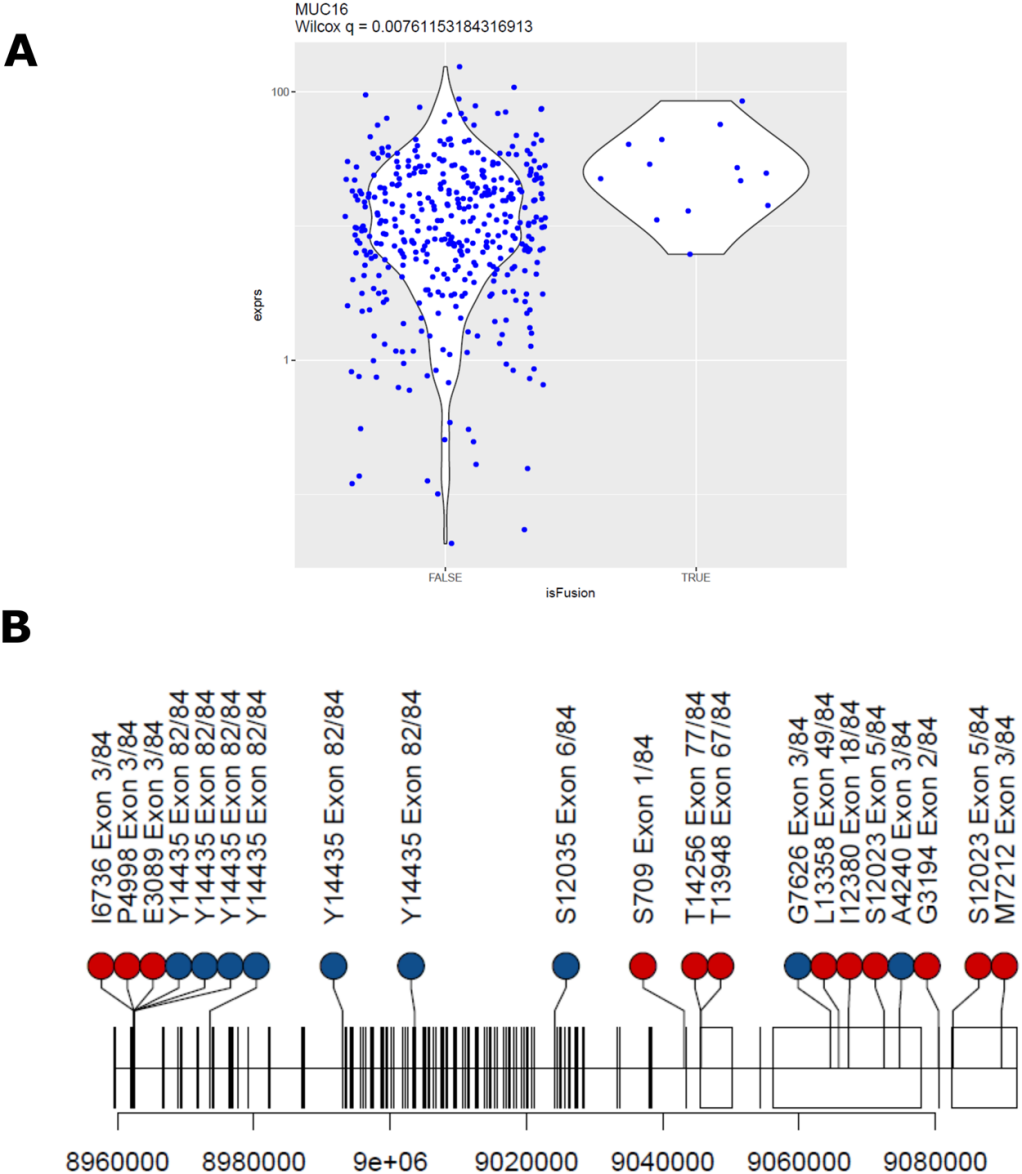
High levels of MUC16 are associated with fusion status. (**A**) Tumors with detected MUC16 fusion transcripts exhibit significantly higher MUC16 expression levels (Wilcox q-value = 0.008). Breakpoint distribution of MUC16 fusion events in primary ovarian transcriptomes, with classifications as outgoing (MUC16 N-terminal; red) or incoming (MUC16 C-terminal; blue). Mapping on to NM_024690 is shown.

Next, we sought to identify genes that exhibit significant expression differences between samples with or without detected fusion transcripts in the same gene. The TCGA primary ovarian tumor transcriptomes yielded 48 genes with this property (Table 1 and Supplementary Table S4), with several known genes exhibiting fusion-associated expression increases at a statistically significant level (Fig. 3). No genes with fusion-associated downregulation were identified, despite using a two-sided Wilcoxon test analysis. A notable gene with elevated expression levels correlated with fusion transcripts is MUC16/CA125, which occurred in 20 TCGA primaries (Fig. 4; Supplementary Table S5). Elevated serum CA125 is correlated with shorter overall survival among ovarian cancers^28^. CA125 fusion-positive primary tumors express the antigen transcript at levels in the upper quartiles of expression when all TCGA transcriptomes are considered. Interestingly, a MUC16 fusion (*MUC16-OR7G2*) was detected in only one cell line transcriptome (OVCAR3) (Supplementary Table S2).

**Table 1:** Genes significantly upregulated with positive fusion status.

Genes	Description	q-value	Fold Change
AQP6	aquaporin 6	2.30E-08	8.91
DAPL1	death associated protein like 1	7.82E-08	6.94
VPREB3	pre-B lymphocyte 3	1.00E-07	2.16
INSL3	insulin like 3	1.96E-06	3.71
KISS1	KiSS-1 metastasis-suppressor	5.90E-05	3.73
AQP5	aquaporin 5	1.30E-04	3.67
C1orf186	chromosome 1 open reading frame 186	1.59E-04	1.80
C22orf31	chromosome 22 open reading frame 31	1.59E-04	15.28
TCL6	T-cell leukemia/lymphoma 6 (non-protein coding)	1.59E-04	9.12
KCTD1	potassium channel tetramerization domain containing 1	1.90E-04	1.66
HKR1	HKR1, GLI-Kruppel zinc finger family member	2.20E-04	1.36
KIAA1683	KIAA1683	2.39E-04	4.13
CRYGC	crystallin gamma C	2.56E-04	126.49
ZNF732	zinc finger protein 732	5.28E-04	2.64
KREMEN1	kringle containing transmembrane protein 1	5.39E-04	2.88
WNT10A	wingless-type MMTV integration site family member 10A	5.40E-04	2.79
DIO3	deiodinase, iodothyronine, type III	5.76E-04	26.20
C2orf48	chromosome 2 open reading frame 48	6.74E-04	2.60
FGF12	fibroblast growth factor 12	7.37E-04	2.53
GOLT1A	golgi transport 1A	8.46E-04	2.22
DAND5	DAN domain family member 5, BMP antagonist	9.66E-04	9.91
RRM2	ribonucleotide reductase M2	9.87E-04	1.81
LRRC69	leucine rich repeat containing 69	1.20E-03	7.85
ATP5I	ATP synthase membrane subunit e	1.24E-03	2.01
C7	complement component 7	1.33E-03	10.87
ARID3C	AT-rich interaction domain 3C	1.33E-03	15.54
BACE2	beta-site APP-cleaving enzyme 2	1.64E-03	2.09
COL6A3	collagen, type VI, alpha 3	2.13E-03	6.51
IGLL5	immunoglobulin lambda-like polypeptide 5	2.28E-03	7.15
SHISA9	shisa family member 9	2.53E-03	8.50
CACNA2D3	calcium channel, voltage-dependent, alpha 2/delta subunit 3	2.53E-03	4.04
DEFB126	defensin beta 126	2.66E-03	11.54
CHCHD10	coiled-coil-helix-coiled-coil-helix domain containing 10	2.70E-03	1.55
MFSD7	major facilitator superfamily domain containing 7	2.70E-03	1.80
JAK3	Janus kinase 3	4.29E-03	1.63
HLA-DRB1	major histocompatibility complex, class II, DR beta 1	4.31E-03	2.56
IRX5	iroquois homeobox 5	5.36E-03	30.66
SIX4	SIX homeobox 4	5.48E-03	2.07
CCDC120	coiled-coil domain containing 120	5.51E-03	3.05
C2orf74	chromosome 2 open reading frame 74	6.34E-03	3.13
KLHL23	kelch like family member 23	6.34E-03	3.78
SLC29A1	solute carrier family 29 (equilibrative nucleoside transporter), member 1	7.38E-03	2.34
MSN	moesin	7.57E-03	1.45
MUC16	mucin 16, cell surface associated	7.68E-03	2.35
KCNIP3	potassium voltage-gated channel interacting protein 3	7.74E-03	5.60
PARD3	par-3 family cell polarity regulator	9.01E-03	1.57
GLTPD2	glycolipid transfer protein domain containing 2	9.01E-03	7.07
NAA15	N(alpha)-acetyltransferase 15, NatA auxiliary subunit	9.82E-03	1.38

Q-values represent FDR corrected p-values of fusion-positive versus fusion-negative expression levels (two-sided Wilcoxon test). Fold change of fusion-positive versus fusion-negative expression levels are shown.

These results indicate that the presence of fusion transcripts for certain genes is associated with expression changes of the genes directly involved in the fusion events. Though RNA-seq data alone cannot distinguish among expression changes arising due to promoter swapping/trapping, rearrangement of regulatory sequences in DNA, or another mechanism altogether, we hypothesized that regional overexpression is a general property linked to fusion expression. In this model, ovarian cancer cells with dormant genes are recruited to regions of activated transcription without addressing whether chimeric transcripts are a cause or effect of the rearrangement event.

**Figure 5:**
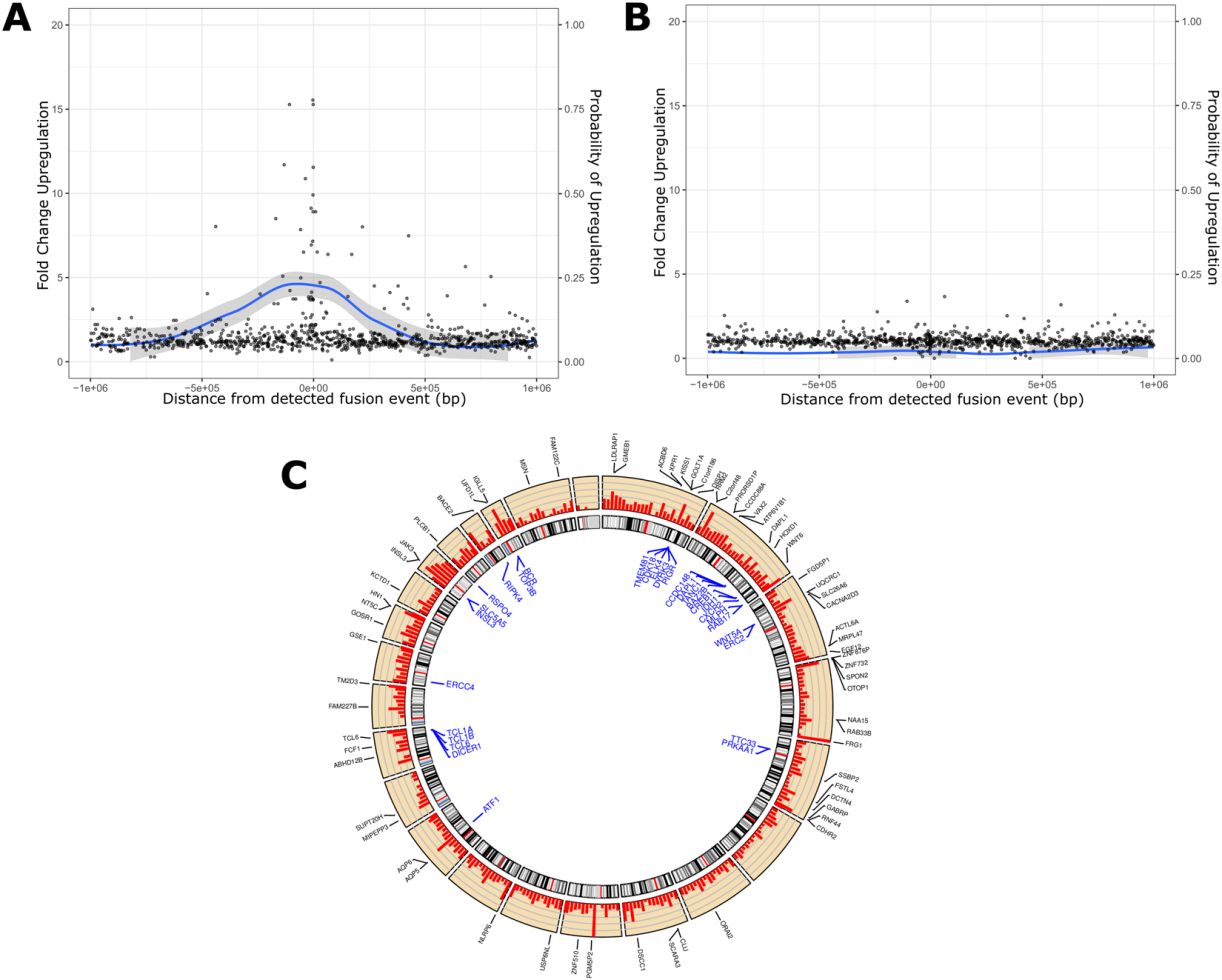
Fusions act as markers of localized expression dysregulation. (**A**) Fusion correlated fold changes of genes in the proximity of fusion genes. Figure illustrates aggregated fold change data for all genes within 1 megabase windows of fusion anchor gene, which lie at the central coordinate (distance = 0). Expected range of fold change (ribbon plot) computed using loess smoothing. (**B**) Identical figure but post-randomization of TCGA expression data and gene fusion status. (**C**) Circos plot indicating selected fusion genes (black), fusion associated dysregulated genes (blue), and ratio of observed vs. expected fusion frequencies (red barplot) across the human genome. Chromosomes 1–22, X, Y presented sequentially in clockwise order from the 12 O’clock position.

To test this hypothesis, we identified genes in a two megabase window centered on each gene involved in a fusion event in the TCGA ovarian data set. These central genes are called ‘anchor genes’. For each anchor gene, we compared the expression level of neighboring genes in TCGA primaries expressing the fusion transcript to those that did not. We found a consistent increase in expression of genes on both sides of anchor genes in primaries that express anchor gene fusions (Fig. 5A). This pattern disappeared when the labels of anchor gene fusion status was randomized (Fig. 5B). These results indicate that the presence of anchor gene fusion transcripts acts as a marker of possible upregulation of nearby genes, an effect that extends to at least one megabase in each direction from the chimeric anchor gene transcript.

Finally, we identified 37 genes in the TCGA data set that were dysregulated specifically when a fusion was detected in one of 78 nearby anchor genes (Fig. 5C and Supplementary Table S6). These associated genes were typically upregulated and consist of a wide repertoire of known cancer associated genes, including *ERCC4, DICER1, BCR, R-spondin 4 (RSPO4), INSL3, ELK3*, and *IDH1*. Dysregulated genes exhibiting fusion associated fold changes greater than 2.13 (>90^th^ percentile of all changes) were analyzed for enrichment using g:Profiler^29^, identifying enrichment in the transport of immune response modulators (*RAB17, PIGR*), peptidyl-serine phosphorylation (*WNT5A, MAST3, TCL1A/B, PRKAA1, CHEK2*), and serine/threonine protein kinase activities (*BCR, MAST3, PRKAA1, CDK18, DYRK3, RIPK4, CHEK2*). Together, these results suggest that selection for regionally upregulated genes in the neighborhoods of structural rearrangement events targets a small number of pathways that are associated with cancer cell fitness.

## Discussion

High-grade serous ovarian cancer has long been known to be a disease characterized by genomic instability and structural variation^30,31^. Our data indicates that all four known serous ovarian cancer subtypes consistently exploit this instability to generate chimeric transcripts, making gene fusions a common aberration in HGSOC.

The large set of gene fusion predictions developed in this study allowed us to examine several characteristics of expressed fusions to infer factors that contribute to fusion creation. Fusion transcripts in HGSOC generally arise through intrachromosomal rearrangements suggesting that localized events, such as tandem duplications, dominate their creation. At the levels of individual genes it appears that biological pressures influence breakpoint positions. In contrast to a simple model in which gene fusion breakpoints occur with uniform probability across genes, fusions from primary tumor transcriptomes were found to arise from breakpoints that tend to occur in UTRs, while breakpoints in coding regions are biased to minimize disruptions to encoded proteins or protein domains (Fig. 2). These results suggest that both ovarian primary samples and cell lines exhibit strong bias against rearrangements that destroy protein coding potential of fusion transcripts.

In addition, the contrast between TCGA primary transcriptomes and cell line observations suggests that there is *in vivo* selection in primary tumors against the generation of fusion transcripts disruption protein coding regions, which could be an effect of selection against events that enhance immunogenicity through the creation neo-antigens. This idea is supported by our observation that cell lines, which are propagated *in vitro* in the absence of immunological surveillance, have higher rates of breakpoints in coding regions than primaries (Fig. 1B). Since breakpoints within coding sequences shuffle protein domains to create novel oncogenic proteins, they also can enhance immunogenicity by creating neo-antigens, as has been previously shown to occur in cancer patients with high somatic mutational loads^32^. Conversely, it is possible that there is positive selection for alterations that change the expression level of one of the genes involved in the fusion without changing its protein coding potential. For example, breakpoints within untranslated regions, which increase the expression of fitness genes could be selected for by evolving cancer cells. Although these events would not be predicted to encode novel proteins with neomorphic properties, they offer an efficient mechanism to place existing coding sequences under the control of novel promoters while reducing the chance of inducing an anti-tumor immune response, suggesting promoter swapping events as a simple fusion event to increase cell fitness. Indeed, the observed enrichment of UTRs in our data suggests that shuffling of regulatory element(s) into apposition with newly associated coding regions is a broadly used mechanism by cancer cells to rewire the expression regulation of genes using structural rearrangements between 20 kilobases downstream and 100 kilobases upstream of affected genes, events that have been reported across 20 cancer types^33^. By using expressed transcripts as evidence of structural rearrangement, our study extends the effect of expression dysregulation to a megabase centered on the fused gene.

Our observation of megabase-scale localized upregulation of genes in the presence of anchoring gene fusion events is consistent with the findings reported by the Pan-Cancer Analysis of Whole Genomes network, which found a pattern of increased gene expression in the neighborhood of rearrangement breakpoints across a broad range of cancers^7^. A suggested mechanism for this phenomenon was interruption of chromatin loops via disruption of topologically-associating domains. The regional dysregulation centered around expressed gene fusions potentially introduces a novel role for fusion transcripts. Chromosomal rearrangements which cause of gene fusions are frequent in human tumors especially highly aneuploidy tumors. However, only a minority of these events are necessarily functional resulting in driver genetic alterations. Chromosomal rearrangements can lead to multiple perturbations within the genome, some of which may be selected for, and others, which may be neutral. Our results provide evidence that some expressed fusion transcripts may be selected for their ability to rewire the transcriptional landscape in the region of the fusion rather than for the enhanced fitness conferred by fusion product itself.

Together, our results demonstrate a potential use for detected fusion transcripts in the interpretation of altered cancer transcriptomes as a signal of underlying structural changes in the genome. We have shown evidence of clustering of overexpressed genes in the neighborhood of some fusions suggesting that heritable genomic rearrangement events contribute to the overexpression of genes in the cluster. The significance of this finding is that coordinated expression changes amongst clustered genes is a common evolutionary feature, for example in both normal development (Hox genes) and also in the evolution of malignancy in cancer cells (HSP1 regulation of genes such as NDRG2 and TPD52^34^). The presence of fusions in cancer transcriptomes are an indicator that nearby gene expression changes should be analyzed carefully for evidence of co-regulation, either by bioinformatics approaches or co-analysis of features in genomic DNA.

The identification of gene fusions which alter the transcription of gene ensembles may inform possible therapeutic modalities, as their expression is specific to diseased cells and their detection implies that cancerous cells exist with a bulk sample. Therefore, gene fusions can function as a flag that evidence of a cell clone carrying a rearrangement exists, genes associated with these clusters can offer a wide variety of known actionable targets in cancer treatments. For example, we observed that PLCB1 fusions were associated with overexpression of R-spondins. Inhibition of the Wnt pathway may be efficacious in triple negative breast cancers overexpressing R-spondins^35^, a therapeutic relationship that should be explored in HGSOC demonstrating the PLCB1 fusion or high R-spondin levels for other mechanistic reasons.

## Conclusions

Our analyses identified positional biases in the breakpoint locations of fused genes. Gene fusion breakpoints within 5′-UTRs and 3′-UTRs are enriched to avoid disruptions of coding regions and breakpoints within coding regions exhibit breakpoints enriched in N-terminal proximal breakpoints. We demonstrate that significant regional overexpression of intact genes in patient transcriptomes can occur within 1 megabase of novel gene fusions, identifying a previously unrecognized mechanism through which cancer cells remodel their transcriptomes and identifying a new way to utilize gene fusions as an indicator of regional expression changes in diseased cells. In summary, we have identified a novel class of gene fusions that perturb the expression of nearby genes that may contribute to the phenotype of HGSOC and establish a new analytical paradigm for analyzing the cancer transcriptome.

## Methods

### Sequencing

RNA from ovarian cancer cell lines was extracted using the RNAeasy Plus kit (Qiagen) yielding RNA samples with RIN numbers (Agilent Bioanalyzer) of 8.0 or greater. Sample libraries were prepared using the TruSeq Stranded mRNA preparation kit (Illumina) and run on Illumina HiSeq2000 instruments generating 2 × 101bp reads at a target library depth of >100 million reads per sample.

### Data Sets

TCGA Ovarian Data for 420 transcriptomes in distribution set phs000178.v7.p6.c1 were obtained under an approved Data Access Request from the National Center for Biotechnology Information Genotypes and Phenotypes Database (NCBI dbGaP). Picard (v1.56) was used to regenerate fastq files from BAM files downloaded from dbGap prior to bioinformatic analysis. Ovarian cancer cell line data is available from the NCBI BioProject portal (https://www.ncbi.nlm.nih.gov/bioproject/) under accession number PRJNA369618 and described further in^36^.

Transcriptomes for seven normal ovarian fallopian tube secretory epithelial cell samples from the Australian Ovarian Cancer Study (AOCS) (AOCS172–178)^4^ were analyzed with defuse (v0.6.1) to compile a list of fusion predictions that were likely to be false positives.

### Expression Data Analysis

Transcriptomes were aligned using Tophat 2.0.3/bowtie 1 with default settings and coverage search turned off. Expression values were computed from the alignments with Cufflinks 2.0.2 with bias and multi-read correction parameters enabled using the UCSC hg19 gene model to produce FPKM values.

### Gene Fusion Predictions

Defuse v0.6.1^16^ was used to predict fusion transcripts from paired fastq files in a parallelized compute environment utilizing Sun Grid Engine and compute nodes with at least 48 gigabytes of available memory for each defuse process and a hg19 based defuse reference dataset. Supporting software included samtools (v0.1.18), bowtie (v.0.12.7), R (v3.0.0), and gmap (v2013-07-20). Defuse predictions for TCGA and cell line transcriptomes with classifier probabilities greater than or equal to the threshold set using ovarian samples (0.81) by McPherson *et al*. and not present in the set of predictions from ovarian fallopian tube secretory epithelial cell samples were used for further analysis. Enrichment statistics on gene fusion features were calculated using a random model that assumes a uniform level of recombination across the entire genomic space.

### Subtype classification of transcriptomes

Transcriptome subtypes were assigned according to the CLOVAR serous ovarian cystadenocarcinoma signatures and the ssGSEA approach previously published^20^. TCGA subtypes were reclassified using the 489 TCGA samples expression table (‘TCGA_489_UE.gct’) obtained from tcga-data.nci.nih.gov. Probe set level expression values were collapsed to gene symbols using the HG-U133A chip definition file (ftp://ftp.broadinstitute.org/pub/gsea/annotations/HG_U133A.chip) prior to scoring subtype signature activation. A reimplemented version of single sample Gene Set Enrichment Analysis (ssGSEA) according to the protocol in Verhaak *et al*. was used to classify cell lines into subtypes. Briefly, normalized enrichment scores were calculated by determining enrichment scores for 1,000 randomly permuted gene lists and computing the mean value of expected raw scores for these permutations. Recalculated raw ssGSEA scores were verified to be correlated across all TCGA tumors correlated each within subtype signature (Data not shown). ssGSEA scores were then computed for cell lines using RNA-seq based expression data to assign each sample to a molecular subtype.

## Supplementary information

Supplementary Dataset 1

## Data Availability

Transcriptome data for ovarian cancer cell lines is available from the NCBI BioProject portal (https://www.ncbi.nlm.nih.gov/bioproject/) under accession number PRJNA480486. Other data sets are publically available; see Methods for details.
